# Thysanoptera of Canada

**DOI:** 10.3897/zookeys.819.26576

**Published:** 2019-01-24

**Authors:** Robert G. Foottit, H. Eric L. Maw

**Affiliations:** 1 Agriculture and Agri-Food Canada, Ottawa Research and Development Centre and Canadian National Collection of Insects, Arachnids and Nematodes, K. W. Neatby Bldg., 960 Carling Ave., Ottawa, Ontario, K1A 0C6, Canada Agriculture and Agri-Food Canada Ottawa Canada

**Keywords:** Barcode Index Number (BIN), biodiversity assessment, Biota of Canada, DNA barcodes, thrips, Thysanoptera

## Abstract

The known Canadian Thysanoptera fauna currently consists of 147 species, including 28 non-native species, and there are five additional species found only indoors. DNA barcoding data, presence of species in adjacent regions, and preliminary evidence of the presence of host-associated cryptic species suggest that there may be as many as 255 additional species awaiting discovery or description in Canada.

The order Thysanoptera (commonly known as ‘thrips’, not to be confused with the genus *Thrips*) is relatively small, with 6200 species known worldwide (Mound 2018). However, the order is most diverse in the tropical zones where the faunas have received relatively little attention; thus many additional species are expected. About 765 species are recorded in North America north of Mexico (unpublished records for the United States collated by Richard zur Strassen and presented in ThripsWiki 2018, plus 16 additional species in Canada based on specimens in the Canadian National Collections of Insects, Arthropods and Nematodes [CNCI]). The number of species of Thysanoptera known from Canada has increased by about 45% (Table 1) since Heming’s (1979) synopsis. One species of each of two small families, Heterothripidae and Merothripidae, have been detected since then, with an additional family, Melanthripidae, currently represented by an unidentified species of *Ankothrips* based on a DNA sequence in the Barcode of Life Data System database (www.boldsystems.org).

**Table 1.** Census of Thysanoptera in Canada.

Taxon^1^	No. species reported in Heming (1979)	No. species currently known from Canada^2^	No. BINs^3^ available for Canadian species	Est. no. undescribed or unrecorded species in Canada	General distribution by ecozone^4^	Information sources^5^
**Suborder Terebrantia**						
Aeolothripidae	13	17	41	30	widespread south of Arctic	Chaisson 1986; CNCI, UASM
Heterothripidae	0	1	0	4	Mixedwood Plains	Chaisson 1986; CNCI
Thripidae	59	85 (20)	192	120	all ecozones including a few species in the southern Arctic	Chaisson 1986; CNCI, UASM, USNM
Merothripidae	0	1	0	0	Mixedwood Plains	CNCI
Melanthripidae	0	0	1	0	Pacific Maritime	BIOUG
**Suborder Tubulifera**						
Phlaeothripidae	30	43 (8)	104	100	all ecozones except Arctic	Chaisson 1986; CNCI, UASM, USNM
**Total**	**102**	**147 (28)**	**338**	**255**		

**^1^**Classification follows Mound (2018). **^2^**Number in parentheses indicates number of non-native species included in total. The totals do not include three species of Thripidae and two species of Phlaeothripidae found only on ornamental plants indoors. **^3^**Barcode Index Numbers, as defined in Ratnasingham and Hebert (2013). **^4^**See figure 1 in Langor (2019) for a map of ecozones. **^5^**Data source collection codens: BIOUG, Biodiversity Institute of Ontario, University of Guelph; CNCI, Canadian National Collection of Insects, Ottawa; UASM, University of Alberta, Strickland Museum; USNM, National Museum of Natural History (Thysanoptera specimens housed at Agricultural Research Service, Beltsville, MD).

Since 1979, there have been several important advances in the knowledge of Canadian thrips diversity. Chasson (1986) made a major contribution to the knowledge of the Canadian fauna, providing keys and descriptions of the genera, and collection data for all species known at that time. She reported 140 identified species, with an additional 42 undetermined or undescribed species, and estimated that 65 more species should occur in this country based on distributions of Thysanoptera in the northern USA. The current total (147 identified established species plus five occurring indoors) is only a marginal increase since Chaisson’s (1986) work.

DNA Barcode Index Numbers (BINs) which give a label to clusters of similar mitochondrial COI sequences (DNA barcodes) correspond approximately to species in many groups (Ratnasingham and Hebert 2013). The relationship between BINs and species concepts has not been critically assessed in Thysanoptera. However, assuming that BIN diversity approximates species diversity (see Ratnasingham and Hebert 2013), derived mainly from untargeted sampling by the Biodiversity Institute of Ontario, University of Guelph, suggests that the number of species sampled could potentially be more than twice the current recognized fauna. There is also preliminary evidence that at least a few species consist of complexes of host-associated lineages (R Foottit and E Maw unpubl. data). Based on the presence of species in adjacent parts of the United States, and on the geographic and taxonomic distribution of unidentified BINs in the DNA barcoding data, we estimate that a further 255 species may eventually be found in Canada (Table 1). However, the Phlaeothripidae, many of which are associated with fungi and decaying wood, are particularly poorly collected, and the estimate of 100 undetected phlaeothripid species in Canada is quite imprecise.

Recent revisions affecting the Canadian fauna have been published for *Thrips* (Nakahara 1994), for *Anaphothrips* (Nakahara 1995), and for *Chirothrips* and related genera (Nakahara and Foottit 2012), including descriptions of new species which occur in Canada (*Thripsaurentulus* Nakahara, *Thripsfallaciosus* Nakahara, *Thripsintricatus* Nakahara, *Chirothripshemingi* Nakahara and Foottit).

Most recent literature on Thysanoptera in Canada treats arising economic issues. The eastern species *Echinothripsamericanus* Morgan became a problem in greenhouses in British Columbia (Opit et al. 1997). The non-native *Taeniothripsinconsequens* (Uzel) was reported as a significant pest on sugar maple (Nystrom and Syme 1994). Old World *Frankliniellaintonsa* (Trybom) has become established in the Fraser Valley of British Columbia (Nakahara and Foottit 2007) and has recently been found in Québec (specimens in CNCI). *Drepanothripsreuteri* Uzel now occurs on grapes in British Columbia (Lowery 2015). Burgess and Weegar (1988) surveyed the Thysanoptera associated with canola crops in Saskatchewan.

This analysis clearly indicates that the Thysanoptera of Canada are understudied. Much of the non-agricultural regions of the country are only sparsely sampled, and the relative knowledge of species occurring in the various provinces and regions is not uniform. The best known region is the province of Alberta (87 species, or about 60% of the species known to occur in Canada; specimens in the Strickland Museum, University of Alberta and CNCI) due primarily to the efforts of Bruce Heming. There are no recent identification tools available for most groups in the order.

## References

[B1] Burgess L Weegar HH (1988) Thrips (Thysanoptera) in canola crops in Saskatchewan. The Canadian Entomologist 120: 815–819. 10.4039/Ent120815-8

[B2] Chaisson H (1986) A synopsis of the Thysanoptera (thrips) of Canada. Lyman Entomological Museum and Research Laboratory Memoir No. 17, 153 pp.

[B3] Heming BS (1979) 36. Thysanoptera. In: Danks HV (Ed.) Canada and its insect fauna.Memoirs of the Entomological Society of Canada No. 108, 349–351. 10.4039/entm111108349-1

[B4] Langor DW (2019) The diversity of terrestrial arthropods in Canada. In: Langor DW Sheffield CS (Eds) The Biota of Canada – A Biodiversity Assessment. Part 1: The Terrestrial Arthropods.ZooKeys 819: 9–40. 10.3897/zookeys.819.31947 PMC6355749 30713431

[B5] Lowery DT (2015) Chapter 5.3 Insect and Mite Pests of Grape. Best Practices Guide for Grapes for British Columbia Growers, Penticton, British Columbia, 27 pp http://www.bcwgc.org/best-practices-guide

[B6] Mound LA (2018) Biodiversity of Thysanoptera. In: Foottit RG Adler PH (Eds) Insect Biodiversity: Science and Society.Volume II. Wiley Blackwell, Chichester, 483–499. 10.1002/9781118945582.ch18

[B7] Nakahara S (1994) The genus *Thrips* Linnaeus (Thysanoptera: Thripidae) of the New World. United States Department of Agriculture, Agricultural Research Service, Technical Bulletin Number 1822, 183 pp.

[B8] Nakahara S (1995) Review of the Nearctic species of *Anaphothrips* (Thysanoptera: Thripidae). Insecta Mundi 9: 221–248.

[B9] Nakahara S Foottit RG (2007) *Frankliniellaintonsa* (Trybom) (Thysanoptera: Thripidae), an invasive insect in North America. Proceedings of the Entomological Society of Washington 109: 733–734.

[B10] Nakahara S Foottit RG (2012) Review of *Chirothrips* and related genera (Thysanoptera: Thripidae) of the Americas, with descriptions of one new genus and four new species. Zootaxa 3251: 1–29.

[B11] Nystrom KL Syme PD (1994) Pear thrips, *Taeniothripsinconsequens* (Uzel) (Thysanoptera: Thripidae) on sugar maple in Ontario. Proceedings of the Entomological Society of Ontario 125: 19–28.

[B12] Opit GP Peterson B Gillespie DR Costello RA (1997) The life cycle and management of *Echinothripsamericanus* (Thysanoptera: Thripidae). Journal of the Entomological Society of British Columbia 94: 3–6.

[B13] Ratnasingham S Hebert PDN (2013) A DNA-based registry for all animal species: the Barcode Index Number (BIN) system. PLoS ONE 8(7): e66213. 10.1371/journal.pone.0066213 PMC3704603 23861743

[B14] ThripsWiki (2018) ThripsWiki – providing information on the World’s thrips. http://thrips.info/wiki/Main_Page [accessed 10 April 2018]

